# Characteristics of Passive Solute Transport across Primary Rat Alveolar Epithelial Cell Monolayers

**DOI:** 10.3390/membranes11050331

**Published:** 2021-04-30

**Authors:** Yong Ho Kim, Kwang-Jin Kim, David Z. D’Argenio, Edward D. Crandall

**Affiliations:** 1 Will Rogers Institute Pulmonary Research Center and Hastings Center for Pulmonary Research, Keck School of Medicine, University of Southern California, Los Angeles, CA 90033-0906, USA; kim.yongho@epa.gov (Y.H.K.); kjkim@usc.edu (K.-J.K.); 2 Division of Pulmonary, Critical Care and Sleep Medicine, Department of Medicine, Keck School of Medicine, University of Southern California, Los Angeles, CA 90033-0906, USA; 3 Department of Biomedical Engineering, Viterbi School of Engineering, University of Southern California, Los Angeles, CA 90089-1111, USA; dargenio@usc.edu; 4 Department of Physiology and Neuroscience, Keck School of Medicine, University of Southern California, Los Angeles, CA 90089-9037, USA; 5 Department of Pharmacology and Pharmaceutical Sciences, School of Pharmacy, University of Southern California, Los Angeles, CA 90089-9121, USA; 6 Department of Pathology, Keck School of Medicine, University of Southern California, Los Angeles, CA 90033-9092, USA; 7 Mork Family Department of Chemical Engineering and Materials Science, Viterbi School of Engineering, University of Southern California, Los Angeles, CA 90089-1211, USA

**Keywords:** paracellular permeability, equivalent aqueous pores, tight junctions, barrier properties, air-blood barrier

## Abstract

Primary rat alveolar epithelial cell monolayers (RAECM) were grown without (type I cell-like phenotype, RAECM-I) or with (type II cell-like phenotype, RAECM-II) keratinocyte growth factor to assess passive transport of 11 hydrophilic solutes. We estimated apparent permeability (*P_app_*) in the absence/presence of calcium chelator EGTA to determine the effects of perturbing tight junctions on “equivalent” pores. *P_app_* across RAECM-I and -II in the absence of EGTA are similar and decrease as solute size increases. We modeled *P_app_* of the hydrophilic solutes across RAECM-I/-II as taking place via heterogeneous populations of equivalent pores comprised of small (0.41/0.32 nm radius) and large (9.88/11.56 nm radius) pores, respectively. Total equivalent pore area is dominated by small equivalent pores (99.92–99.97%). The number of small and large equivalent pores in RAECM-I was 8.55 and 1.29 times greater, respectively, than those in RAECM-II. With EGTA, the large pore radius in RAECM-I/-II increased by 1.58/4.34 times and the small equivalent pore radius increased by 1.84/1.90 times, respectively. These results indicate that passive diffusion of hydrophilic solutes across an alveolar epithelium occurs via small and large equivalent pores, reflecting interactions of transmembrane proteins expressed in intercellular tight junctions of alveolar epithelial cells.

## 1. Introduction

It is well recognized that the mammalian lung alveolar epithelium is tightly organized to prevent the excessive passive flow of water and solutes between the interstitial/vascular and alveolar spaces in the lung [1,2]. Passive transport properties of alveolar epithelium are thought to be governed primarily by the tight junctions between adjacent pneumocytes [3,4]. Characterization of tight junctions is essential for understanding the barrier properties of alveolar epithelium [3,5]. It is generally accepted that tight junctions contain aqueous pores whose properties (e.g., size, number, permeability, and selectivity) govern passive transport of hydrophilic solutes (including water and small inorganic ions) across epithelial barriers [6]. Although electrical resistance, ion permeability and strand numbers of tight junctions appear to be widely different in various epithelial models studied under different experiment conditions [7,8,9,10,11], equivalent aqueous pores appear to be in the range of 0.4–0.5 nm radius.

The mammalian lung alveolar epithelium is comprised of type I and type II cells. Alveolar epithelial type I (AT1) cells are thin large flat cells (as compared to type II (AT2) cells) that make up >95% of alveolar surface area. AT2 cells are smaller cuboidal cells that occupy the remainder of the alveolar surface. Although morphological and functional characteristics of AT1 and AT2 cells have been relatively well documented, characterization of tight junctions between these cells has not yet been fully investigated, in part because of incomplete information on various integral membrane proteins (e.g., occludin, claudins and tricellulins) at alveolar epithelial tight junctions. Expression of differences in claudins in particular might affect aqueous pores in tight junctions, leading to possible differences in transepithelial electrical resistance and/or paracellular permeability of hydrophilic solutes (including water and ions), although interactions among claudin expression, electrical resistance and permeability remain in need of further exploration [12,13].

Primary cultured rat alveolar epithelial cell monolayers (RAECM) that exhibit morphological and phenotypic traits of in vivo AT1 and AT2 cells are useful models for investigation of biological/functional characteristics of the alveolar epithelium [14]. It is generally accepted that primary cultured rat AT2 cells transdifferentiate into an AT1 cell-like phenotype—monolayers of which are here called RAECM-I [15] and transdifferentiation toward the AT1 cell-like phenotype can be prevented/reversed by treatment with keratinocyte growth factor (KGF) resulting in AT2 cell-like monolayers here called RAECM-II [16,17,18,19,20]. Both RAECM-I and -II exhibit high transepithelial electrical resistance (*R_t_* > 2 kΩ·cm^2^) with well-formed tight junctions and active ion transport (exhibiting equivalent short circuit current, *I_eq_*, up to 6 μA/cm^2^), consistent with the expected tight barrier properties of the alveolar epithelium in vivo.

Although we previously reported passive transport properties using excised amphibian lung [21] and isolated perfused rat lung [1,22] models, additional studies that address specifically mammalian lung alveolar epithelial passive permeation of hydrophilic solutes are needed. In this study, we investigated passive solute permeability characteristics of tight junctions in both RAECM-I and -II by measuring apparent permeability coefficients (*P_app_*) for 11 hydrophilic solutes (with molecular radius ranging from 0.19 to 2.29 nm) in the apical-to-basolateral direction. Assuming that passive restricted diffusion of these solutes takes place via equivalent water-filled pores residing in tight junctions, observed *P_app_* data were analyzed to yield the radius and number of equivalent water-filled pores. In addition, by treating RAECM-I or -II with 2 mM ethylene glycol-bis(β-aminoethyl ether)*N,N,N′,N′*-tetraacetic acid (EGTA), we investigated effects of perturbation of tight junctions by Ca^++^ depletion on equivalent pore characteristics. Results indicate that both RAECM-I and -II exhibit heteropore (i.e., small and large equivalent pores) characteristics with total equivalent pore area dominated by small equivalent pores; the number of small and large equivalent pores in RAECM-II are greater than those in RAECM-I; and paracellular diffusion of larger hydrophilic solutes is markedly increased in both RAECM-I and -II after EGTA exposure.

## 2. Materials and Methods

### 2.1. Primary Cultured Rat Alveolar Epithelial Cell Monolayers (RAECM)

The detailed procedure for generation of freshly isolated rat type II cells has been described elsewhere [15,16,17]. The animal protocol involving usage of rats was approved by the Institutional Animal Care and Use Committee of the University of Southern California. Briefly, fresh type II pneumocytes were isolated from adult male, specific pathogen-free, Sprague-Dawley rats (125–150 g) using elastase digestion and enriched by panning the crude cell population on rat immunoglobulin G-coated Petri dishes. Enriched type II cells were then plated onto porous (0.4 μm diameter) tissue culture-treated polycarbonate filters (12 mm diameter, Transwell, Corning-Costar, Cambridge, MA, USA) at 1.2 × 10^6^ cells/cm^2^ on day 0. For primary culture of RAECM, we used a culture medium (MDS) comprised of a 1:1 mixture of Dulbecco’s minimal essential medium (DMEM) and Ham’s F-12 (Sigma, St. Louis, MO, USA), 0.1 mM nonessential amino acids (Sigma), 0.2% primocin (InvivoGen, San Diego, CA, USA), 10 mM N-(2-hydroxyethyl) piperazine-N′-(2-ethanesulfonic acid) hemisodium salt (HEPES, Sigma), 1.25 mg/mL bovine serum albumin (BSA, BD Bioscience, San Jose, CA, USA), 2 mM L-glutamine (Sigma) and 10% newborn bovine serum (NBS, Omega, Tarzana, CA, USA). Cells were maintained at 37 °C in a humidified atmosphere of 5% CO_2_ + 95% air. Confluent monolayers formed by day 3 in primary culture exhibit AT1 cell-like morphology and phenotype (RAECM-I). For RAECM-II, freshly isolated type II pneumocytes were cultivated using MDS further supplemented with 10 ng/mL KGF (R&D Systems, Minneapolis, MN, USA). Monolayers were fed with a medium without KGF for RAECM-I and with 10 ng/mL KGF for RAECM-II every other day starting on day 3. Volumes of apical and basolateral fluids were 0.5 and 1.5 mL, respectively. We generally used RAECM-I and -II on day 5 or 6 in culture. These monolayers were utilized for determination of possible differences in passive transport properties (i.e., theoretical/equivalent/conceptual heteropore characteristics across the two types of RAECM, despite possible differences in properties such as cell number per unit area.

### 2.2. Bioelectric Properties of RAECM

Transmonolayer resistance (*R_t_*_,_ kΩ·cm^2^) and potential difference (*PD*, mV) were measured using a Millicell-ERS device (Millipore, Bedford, MA, USA) before and at the end of permeability experiments. Background *PD* and *R_t_* were determined using blank filters with appropriate culture fluids and were used to correct measured *PD* and *R_t_*. Equivalent short circuit current (*I_eq_*, μA/cm^2^) was calculated as the ratio between *PD* and *R_t_* after both were background-corrected. *R_t_* is an index of integrity and *I_eq_* is an index of net active ion transport of RAECM-I or -II.

### 2.3. Hydrophilic Solutes

Formamide, acetamide, ethylene glycol, glycine, arabinose, mannitol, and sucrose were purchased as ^14^C-labeled compounds from American Radiolabeled Chemicals (St. Louis, MO, USA). 5-Carboxyfluorescein, sulforhodamine B and fluorescein isothiocyanate (FITC)-labeled 4 kDa and 10 kDa dextrans were purchased from Sigma Chemical (St. Louis, MO, USA).

### 2.4. Measurements of Flux (J) of Hydrophilic Solutes

Apical-to-basolateral fluxes of various hydrophilic solutes across RAECM-I and -II were measured by adding (at *t* = 0) either a ^14^C labeled solute (at 0.1 µCi/mL) or fluorescently labeled solute (at 0.1–1 mg/mL) to upstream (apical) fluid, followed by monitoring radioactivity or fluorescence appearing in downstream (basolateral) fluid by sampling for up to 3 h for radiolabeled solutes and 6 h for fluorescent solutes at 37 °C. After sampling, an equal volume of fresh culture medium without KGF (for RAECM-I) or that supplemented with 10 ng/mL KGF (for RAECM-II) was added to the basolateral compartment in order to maintain constant volume throughout the flux measurement period. To minimize nonspecific adsorption of labeled solutes into the cells or filter inserts/walls of apical and basolateral compartments, 1000-fold excess unlabeled solutes were added to apical and basolateral fluids prior to flux measurements. Radioactivity or fluorescence was assayed using a beta counter (Beckman Instruments, Fullerton, CA, USA) or fluorometer (SpectraMax M2, Molecular Devices, Sunnyvale, CA, USA), respectively. Excitation/emission wavelengths of 490/520 nm were used for 5-carboxylfluorescein and FITC-labeled dextrans, while 565/586 nm settings were utilized for sulforhodamine B. Upstream concentration of labeled solutes was similarly assessed by apical sampling (20 µL) at the beginning and end of each flux experiment. At the beginning and end of the flux experiments, *PD*, *R_t_* and *I_eq_* were assessed to monitor changes in RAECM-I or -II bioelectric properties.

### 2.5. Estimation of Apparent Permeability Coefficients (P_app_)

We estimated *P_app_* from the steady-state flux of a given solute. Hydrophilic solute flux (*J*) is given by *J* = (*V · C*)/(*S ·* Δ*t*), where *V* is basolateral fluid volume, *S* is nominal monolayer surface area (1.13 cm^2^), and *C* is hydrophilic solute concentration in basolateral fluid at Δ*t* (which represents the time interval for assessment of the solutes appearing in downstream fluid). *P_app_* then is estimated as *J/C_o_*, where *J* is the steady-state flux and *C_o_* the hydrophilic solute concentration in apical fluid at *t* = 0.

### 2.6. Effects of EGTA

To investigate the effects of perturbing tight junctions of RAECM-I or -II on equivalent pore characteristics, RAECM-I or -II were pre-treated for 30 min with EGTA at 2 mM in both apical and basolateral fluids, followed by measurements of hydrophilic solute fluxes for up to 3 h for radiolabeled solutes and 6 h for fluorescent solutes with an upstream concentration of 0.06 µCi/mL and 0.1 (or 1) mg/mL, respectively. After each downstream sampling for flux measurements, an equal volume of culture medium (for RAECM-I) or that supplemented with 10 ng/mL KGF (for RAECM-II), each containing 2 mM EGTA, was added back to keep EGTA concentration and volume in downstream fluid constant. Bioelectric properties (*PD*, *R_t_* and *I_eq_*) of monolayers were assessed at the beginning and end of these experiments.

### 2.7. Unstirred Layer Thickness

Unstirred fluid layers residing at the cell membrane-liquid interface in the apical fluid and basolateral fluid can lead to underestimation of *P_app_*. In order to correct for such unstirred layer effects on observed *P_app_*, the thickness of unstirred aqueous layers was estimated from observed *P_app_* of the lipophilic solute benzyl alcohol. Briefly, RAECM-I or -II were apically exposed to 0.1 nCi/mL ^14^C-benzyl alcohol (American Radiolabeled Chemicals), and samples were taken from apical or basolateral fluid at 0, 0.5 and 1 h. The thickness of the sum of apical and basolateral unstirred layers (*δ*) was estimated by 1/*P_app, benzyl alcohol_* = *δ/D_benzyl alcohol_*, where *P_app, benzyl alcohol_* is the apparent permeability coefficient of benzyl alcohol and *D_benzyl alcohol_* is the free diffusion coefficient of benzyl alcohol at 37 °C in water (1.19 × 10^−5^ cm^2^/s, [23]), under the assumption that benzyl alcohol diffusion is limited by unstirred layers existing at both apical and basolateral fluids (but not by apical or basolateral cell membranes) [24]. The estimated unstirred layer thickness was used to correct observed *P_app_* of hydrophilic solutes across RAECM-I or -II, without or with EGTA, using the relation 1/*P_app(obs)_* = 1/*P_app(cor)_* + *δ/D_solute_*, where *P_app(obs)_* is the observed *P_app_*, *P_app(cor)_* the corrected *P_app_*, *δ* the unstirred layer thickness, and *D_solute_* the diffusion coefficient of the corresponding solute. Hereafter, *P_app_* shall denote the corrected *P_app_*.

### 2.8. Calculation of Molecular Radius of Solutes

Molecular weight (*Mw*), free diffusion coefficient (*D*) and molecular radius (*r*) of hydrophilic solutes utilized are listed in Table 1. Molecular radius of solutes was calculated based on their physiochemical properties. For example, if the solute molecular weight is close to the molecular weight of the medium (e.g., small molecules of formamide through sucrose), solute radius was averaged for values using the two relations of (i) *r* = (3 · *Mw*)/(4 · *π* · *ρ* · *N*)^1/3^, where *ρ* is the density of solute and *N* is Avogadro’s number, and (ii) *r* = (*k · T)/*(6*·π · η · D*), where *k* is the Boltzmann constant, *T* is absolute temperature and *η* is viscosity of medium (i.e., water). We chose to use the averaged radius from the two equations (i.e., (i) and (ii) above) because the use of either equation (i) or (ii) alone for solute radius estimation can lead to over- or under-estimation [25]. For polydisperse solutes (e.g., dextrans), the empirical equation *r* = 0.33*·*(*Mw*)^0.463^ was used to calculate molecular radius [26].

### 2.9. Equivalent Pore Analysis

Equivalent pore characteristics (i.e., equivalent pore radius and equivalent pore area) of RAECM-I or RAECM-II, without or with 2 mM EGTA, were assessed assuming the following model relating *P_app_* to the radius of hydrophilic solutes (*r*) corrected for unstirred layer effects using the relation [8,25,34]:

(1) $$P_{app}\left(r\right)=\frac{D}{s}\times \frac{A_{p}}{dx}\times f\left(\frac{r}{R}\right)$$

where *D* is the free diffusion coefficient of the hydrophilic solute of radius *r* at 37 °C in water, *S* is nominal surface area of the monolayer (of 1.13 cm^2^), *dx* is equivalent pore length, *r* is solute molecular radius (Table 1), *R* is equivalent pore radius (assuming water-filled cylindrical equivalent pores), and *A_p_* is total equivalent pore area determined by *R* and the number of equivalent pores *N*. Pore length *dx* is assumed to span the entire depth of the cell–cell adjoining region where tight junctions are located. Accordingly, *dx* was set as the thickness of RAECM-I and -II of 0.5 and 4.2 µm, respectively, as previously reported [35]. The function *f*(*r/R*) is defined [8,25,34] as:

(2) $$f\left(\frac{r}{R}\right)=\frac{{\left[1-\left(\frac{r}{R}\right)\right]}^{2}[1-2.105\left(\frac{r}{R}\right)+2.0805{\left(\frac{r}{R}\right)}^{3}-1.7068{\left(\frac{r}{R}\right)}^{5}+0.72603\left(\frac{r}{R}{)}^{6}\right]}{[1-0.7589\left(\frac{r}{R}{)}^{5}\right]}$$

Observed apparent solute permeability *P_app_* vs. solute radius for hydrophilic solutes appears to exhibit heteropore characteristics (i.e., one population of small equivalent pores for restricted diffusion of formamide through mannitol and another population of large equivalent pores for passive diffusion of sucrose through 10 kDa dextran) for the dataset of RAECM-I. These heteropore characteristics can be described as follows, assuming the length of small and large equivalent pores are equal:

(3) $$\begin{array}{cc} P_{app}\left(r\right)=\frac{D}{s\cdot d_{x}}\cdot A_{p,l}\cdot f\left(\frac{r}{R_{l}}\right), & r\geq r_{ref}\\ P_{app}\left(r\right)=\frac{D}{s\cdot d_{x}}\cdot A_{p,s}\cdot f\left(\frac{r}{R_{s}}\right)+\frac{D}{s\cdot d_{x}}\cdot A_{p,l}\cdot f\left(\frac{r}{R_{l}}\right), & r<r_{ref} \end{array}$$

where *D*, *S* and *dx* are as defined above, while *R_s_* and *R_l_* represent the equivalent pore radii of small and large equivalent pores, and *r_ref_* is the radius the reference solute. In Equation (3), *A_p,s_* and *A_p,l_* denote the total small and large equivalent pore areas, determined by their respective radii (*R_s_* and *R_l_*) and equivalent pore numbers (*N_s_* and *N_l_*). Equation (3) was used together with the apparent solute permeability *P_app_* vs. solute radius measurements to estimate the unknown parameters (*R_l_, N_l_ R_s_* and *N_s_*) via nonlinear regression. Estimation of these parameters was performed with the ADAPT software [36], using a maximum likelihood approach assuming normally distributed errors with additive and proportional error variance. It is noted that the reference solute for other datasets appears to be smaller in size than sucrose used for the RAECM-I dataset. Because the pore theory model used (Equation (2)) is empirical, it is not unexpected that the reference solute radius between small and large pores would be different depending on cell type. In the model estimation process, the reference solute radius was therefore selected to yield best fits to the dataset. Therefore, we compared the estimation results using some different values of *r_ref_*. Final estimation results were based on comparing goodness of fit (log likelihood values) and considering standard errors of the estimated parameters.

### 2.10. Data Analysis

Data are presented as mean ± standard deviation (n), where n is number of observations. Student’s unpaired *t*-tests were used for comparisons of bioelectric properties and unstirred layers between RAECM-I and -II. Three-way analysis of variance (ANOVA) was performed using Prism (GraphPad, San Diego, CA) on *P_app_* of RAECM-I and RAECM-II in the absence and presence of EGTA to discern *P_app_* differences between RAECM-I and RAECM-II and effects of EGTA on *P_app_*. Post-hoc multiple comparisons for all *P_app_* were performed based on Tukey’s procedure with statistical significance set at *p* < 0.05.

## 3. Results

*R_t_* and *I_eq_* of RAECM-I at baseline were 2.94 ± 0.64 kΩ·cm^2^ and 4.42 ± 0.77 μA/cm^2^, respectively (n = 77). These monolayers were used to measure permeability of various hydrophilic solutes with and without EGTA treatment (n = 31). RAECM-I did not show significant changes in *R_t_* or *I_eq_* during the permeability measurement period for up to 3 h in the absence of EGTA. *R_t_* and *I_eq_* of RAECM-I (n = 46) fell by ~90% and ~100%, respectively, after up to 3 h permeability measurements in the presence of EGTA.

*R_t_* and *I_eq_* of RAECM-II at baseline were 3.05 ± 0.94 kΩ·cm^2^ and 6.66 ± 1.56 μA/cm^2^, respectively (n = 82). *I_eq_* in RAECM-II was significantly increased compared to that in RAECM-I (*p <* 0.0001) without any significant difference in *R_t_* (*p* = 0.3927). *R_t_* and *I_eq_* of RAECM-II did not change significantly during 3 h of permeability measurements in the absence of EGTA. Similar to observations in RAECM-I, *R_t_* and *I_eq_* of RAECM-II fell by ~95% and ~100%, respectively, after 3 h of permeability measurements in the presence of EGTA (n = 42).

The molecular radius and diffusion coefficient of 11 hydrophilic solutes are shown in Table 1. Because the measured *P_app_* are comprised of the true *P_app_* across cell monolayers and across hydrodynamic boundary layers residing at the apical and basolateral sides (i.e., unstirred layers), we estimated unstirred layer thickness in our system and corrected all measured *P_app_* to estimate true *P_app_* for the solutes studied. The thicknesses of unstirred layers (i.e., as a sum of unstirred layers adjacent to apical and basolateral sides of RAECM-I and -II), deduced from the observed *P_app_* of ^14^C-benzyl alcohol (6.51 ± 0.67 × 10^−5^ cm/s in RAECM-I and 6.09 ± 1.03 × 10^−5^ cm/s in RAECM-II), was 1.84 ± 0.17 mm in RAECM-I and 1.99 ± 0.36 mm in RAECM-II, respectively, but the difference was not significant (*p* > 0.05) (Table 2). It can be noted that the unstirred layer thickness obtained in our experiments is inclusive of the nominal thickness of 10 µm for the filter membrane on which the RAECM were cultured. The maximum correction for unstirred layer effects on *P_app_* was 52.48% for glycine across RAECM-II with EGTA and 12.09% for acetamide across RAECM-II without EGTA, while for other *P_app_* the correction was smaller with the smallest correction of 0.02% for *P_app_* of FITC-4 kDa dextran across RAECM-II without EGTA.

*P_app_* (after correction for the unstirred layer effects) of the solutes across RAECM-I or -II, without and with 2 mM EGTA, in the apical-to-basolateral direction are listed in Table 3a. As expected, *P_app_* decreases with increased solute molecular weight. Significant differences in *P_app_* of solutes across RAECM-I (or -II) without and with EGTA were observed (Table 3 and Table A1 and Table A2 in Appendix A).

**Figure 1:**
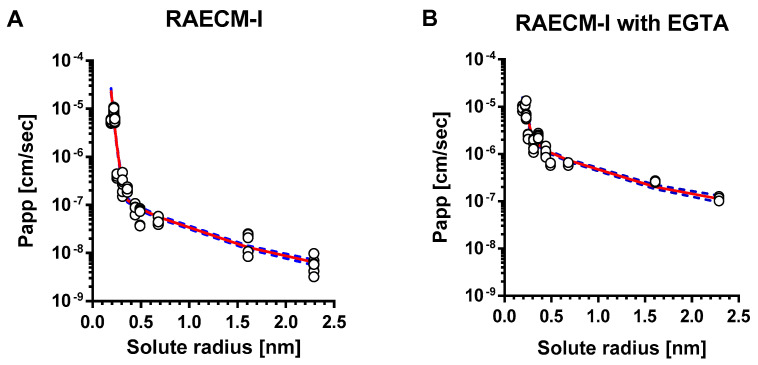
Heteropore analysis of *P_app_* across RAECM-I. *P_app_* across RAECM-I in the absence (**A**) and presence (**B**) of EGTA were analyzed using a maximum likelihood optimization approach, yielding small and large equivalent pore characteristics shown in Table 4. Circles represent individual *P_app_*, and the solid curve in red denotes the composite equivalent pore characteristics that best fit the observed *P_app_* versus solute radius data. Blue dotted curves represent solid red curve ± standard errors. Figure A1 in Appendix A shows the relationship between mean values of *P_app_* and solute size, similar to that shown above in Figure 1 (based on optimization using all entries of *P_app_*).

The relationships between *P_app_* of hydrophilic solutes across RAECM-I and solute radius are shown in Figure 1 for RAECM without (Figure 1A) and with (Figure 1B) EGTA. *P_app_* of hydrophilic solutes across RAECM-I increased by up to 19.95 times with EGTA. For mannitol through FITC-10 kDa dextran, the increases were >9.98. The unbroken lines in Figure 1 represent the best-fit relationship between all individual data points of *P_app_* across RAECM-I (at baseline and with EGTA, respectively) and solute radius, obtained by maximum likelihood approach. Dotted lines in Figure 1 represent the upper and lower error limits for the best fit curves. As seen, the heteropore characteristics estimated by the two methods (i.e., based on using all entries of individual *P_app_* and using mean values of *P_app_* in general) yielded similar results for the number of equivalent pores and equivalent pore radii, albeit the standard error associated with each parameter estimation tends to be larger in the method based on using mean values of *P_app_*. In essence, *P_app_* data for RAECM-I at baseline are best described by the presence of two populations of small and large water-filled cylindrical equivalent pores.

As summarized in Table 4, equivalent pore analysis yielded small and large equivalent pore radii in RAECM-I at baseline of 0.32 nm and 11.56 nm, respectively. The numbers for small and large equivalent pores are 9.15 × 10^11^ and 1.88 × 10^5^, respectively (Table 4), which account for 99.97% and 0.03% of respective total equivalent pore area (Table 5). These results suggest that passive restricted diffusion of hydrophilic solutes across RAECM-I predominantly takes place via small equivalent pores of 0.32 nm radius. EGTA treatment of RAECM-I increased the small and large equivalent pore radii by 1.84 and 1.58 times (Table 4), respectively, and decreased the small equivalent pore number by 80.26 times and caused concomitant decrease in associated fractional equivalent pore area for small equivalent pores by 7.06% (Table 5), which might be indicative of the dilation and/or fusion of small equivalent pores to form equivalent pores of increased size due to EGTA treatment.

**Figure 2:**
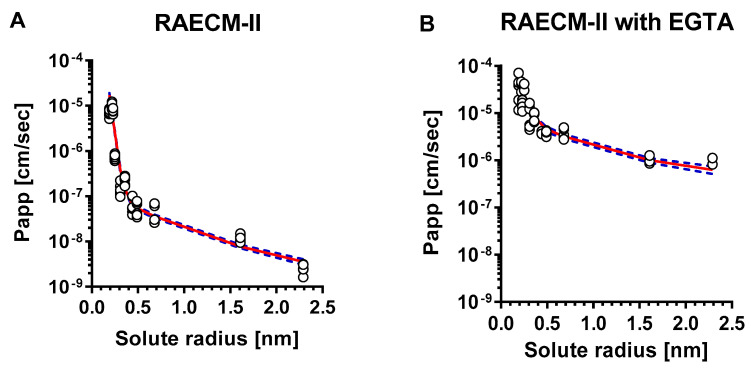
Heteropore analysis of *P_app_* across RAECM-II. *P_app_* across RAECM-II in the absence (**A**) and presence (**B**) of EGTA were analyzed using a maximum likelihood optimization approach, yielding small and large equivalent pore characteristics shown in Table 6. Circles represent individual *P_app_*, and the solid curve in red denotes the composite equivalent pore characteristics that best fit the observed *P_app_* versus solute radius data. Blue dotted curves represent solid red curve ± standard errors. Figure A2 in Appendix A shows the relationship between mean values of *P_app_* and solute size, similar to that shown above in Figure 2 (based on optimization using all entries of *P_app_*).

The relationship between *P_app_* of hydrophilic solutes across RAECM-II and solute radius is depicted in Figure 2A (at baseline) and Figure 2B (with EGTA treatment). The unbroken lines in Figure 2 represent the best-fit relationship between the *P_app_* for all individual data points for RAECM-II (at baseline and with EGTA, respectively) and solute radius, obtained by maximum likelihood approach. Dotted lines in Figure 2 represent the upper and lower error limits for the best-fit curves represented by unbroken lines. As seen, *P_app_* obtained for RAECM-II at baseline (Figure 2A) are best described assuming the existence of two equivalent pore populations with radii of small and large equivalent pores of 0.41 nm and 9.88 nm, respectively, similar to those found in RAECM-I (Figure 1A). The number of small equivalent pores in RAECM-II at baseline were much smaller (0.11 times) at 1.07 × 10^11^, whereas the number of large equivalent pores in RAECM-II at baseline exhibited 1.45 × 10^5^, respectively (i.e., 0.77 times that estimated for RAECM-I at baseline) (Table 4 and Table 6). Passive restricted diffusion of hydrophilic solutes across RAECM-II at baseline appears to occur predominantly via small equivalent pores with radius of ~0.41 nm occupying 99.92% of total equivalent pore area (Table 7). EGTA treatment increased *P_app_* of all hydrophilic solutes across RAECM-II, leading to increased small equivalent pore radius by 1.90 times to 0.78 nm and large equivalent pore radius by 4.34-fold to 42.89 compared to those in RAECM-II at baseline. In the presence of EGTA (Table 5 and Table 7), the number of small equivalent pores decreased from 1.07 × 10^11^ to 0.785 × 10^11^, while the small equivalent pores only occupy 82.71% of total equivalent pore area in RAECM-II with EGTA, a larger change than that seen in RAECM-I following EGTA treatment.

**Figure A1:**
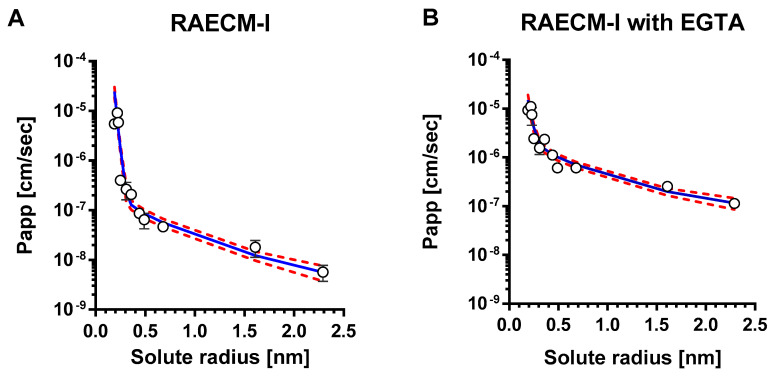
Mean values of *P_app_* versus corresponding solute radius across RAECM-I in the absence (**A**) and presence (**B**) of EGTA were analyzed using a maximum likelihood optimization approach, yielding small and large pore characteristics shown in Table A3. Circles represent mean *P_app_* and solid curve in blue denotes the heteropore characteristics that best fit the observed mean *P_app_* versus corresponding solute radius data. Standard deviation for each mean *P_app_* is also shown. Red dotted curves represent solid blue curve ± standard errors.

**Figure A2:**
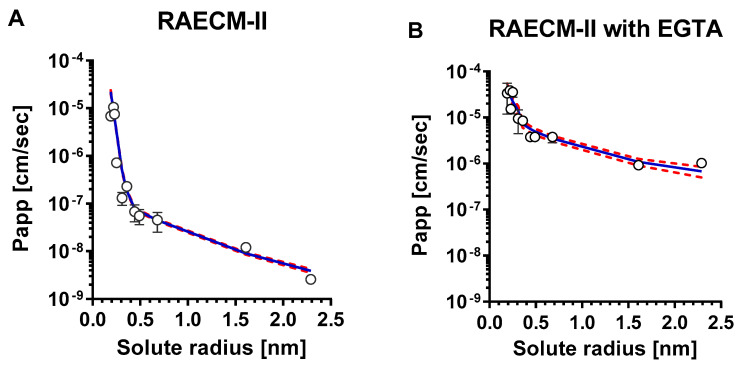
Mean values of *P_app_* versus corresponding solute radius across RAECM-II in the absence (**A**) and presence (**B**) of EGTA were analyzed using a maximum likelihood optimization approach, yielding small and large equivalent pore characteristics shown in Table A5. Circles represent mean *P_app_* and solid curve in blue denotes the heteropore characteristics that best fit the observed mean *P_app_* versus corresponding solute radius data. Standard deviation for each mean *P_app_* is also shown. Red dotted curves represent solid blue curve ± standard errors.

It should be noted here that Table A3, Table A4, Table A5 and Table A6 and Figure A1 and Figure A2 in the Appendix A are based on maximum likelihood optimization using mean values of *P_app_* for analyses of equivalent pore characteristics. In general, usage of mean *P_app_* for heteropore analyses yields larger standard errors for estimated parameters than usage of all data, so we did not use the mean values for further comparisons or discussion. The Appendix A contains these results to demonstrate completeness of our analyses.

## 4. Discussion

Both RAECM-I and -II are characterized by two (i.e., heteropore) populations of small and large equivalent pores. Radii of small and large equivalent pores in RAECM-I are similar to those of respective equivalent pore populations in RAECM-II. EGTA treatment led to a significantly increased *P_app_* and number of large equivalent pores in both RAECM-I and especially RAECM-II, indicating that EGTA affects primarily the large equivalent pore populations in RAECM-I and -II.

An equivalent pore radius of 0.4–0.5 nm has been reported for several in vitro cell monolayer models, including primary cultured rat type I-like pneumocytes [37], Madin-Darby canine kidney (MDCK) cells [9,10], Caco-2 cells [9,10,11,38] and T84 cells [11]. Although small and large equivalent pore characteristics of RAECM-I monolayers were reported previously [39,40], their results were weakened by use of an inadequate number of hydrophilic solutes of 0.2–0.8 nm radius. Similarly, another study [37] reported a small equivalent pore radius in rat AEC monolayers of ~0.5 nm, but a large equivalent pore radius was not estimated due to the fact that they used only mannitol and urea, which have solute radii of 0.3–0.4 nm. Similarly, polyethylene glycol (PEG) oligomers of 0.3–0.7 nm radius have been used to estimate small equivalent pore characteristics but not large equivalent pore characteristics in MDCK cells [10]. In this study, we utilized 11 hydrophilic solutes with a range of radii from 0.2 nm to 2.3 nm in order to determine small and large equivalent pore characteristics in both RAECM-I and RAECM-II at baseline and in the presence of EGTA.

Compared to other rat AEC monolayer equivalent pore studies cited above [37,41], which utilized monolayers with much lower *R_t_* than our RAECM (average *R_t_* > 2 kΩ·cm^2^), low *R_t_* potentially leads to much greater *P_app_* and larger equivalent pore characteristics. At baseline, the small equivalent pore radius of RAECM-I or -II is 0.31 or 0.42 nm, consistent with the reported equivalent pore radius in other epithelial barrier models [9,10,11,37,38]. Small equivalent pores in RAECM-I and -II occupy >99% of total equivalent pore area, in good agreement with Cavanaugh et al. [39], where RAECM-I was modeled with a small equivalent pore population whose radius is 0.43 nm occupying >99% of total equivalent pore area. In earlier studies of passive solute diffusion across other epithelial barriers, including rat small intestine [42], toad skin [43] and toad bladder [44], similar radii for small and large equivalent pores were reported, although the numbers of equivalent pores varied widely. Regarding the large equivalent pore population, we found an equivalent pore radius of ~9–12 nm for RAECM-I or -II at baseline conditions, in good agreement with our previous report based on restricted diffusion of various dextran molecules across RAECM-I [45].

The small and large equivalent pore populations found in our studies are in reasonable agreement with several ex vivo studies. Fluid-filled mammalian or amphibian lungs studied in our laboratory [1,8] and a dog lung study [46] have demonstrated that tight junctional equivalent pores in alveolar epithelium of ex vivo lungs can be characterized by a predominant population of small equivalent pores of ~0.5 nm radius and a few large equivalent pores of ~5–8 nm radius. In this context, unlike the agreement in estimated small equivalent pore radius of RAECM, there is a discrepancy in the estimated large equivalent pore radius between the studies. For example, Dodoo et al. [40,41] reported a large equivalent pore radius of 22 nm, whereas Cavanaugh et al. [39] estimated a large equivalent pore radius of ~4 nm. These discrepancies between our findings and others may have resulted, in part, from an inadequate range of hydrophilic solutes of 0.4–0.8 nm radius to estimate large equivalent pore characteristics used in these latter studies. Despite the difference in large equivalent pore radii among these studies, which may be due to variation in experimental procedures, there is agreement that the large equivalent pores occupy only a small percentage of total equivalent pore area.

Several studies utilizing a reliable in vitro AT2-like cell monolayer model [35,47] have been previously reported. For example, KGF-treated primary rat AT2 cell monolayers (RAECM-II) exhibit AT2 cell-like morphology (i.e., cuboidal shape) and phenotype (i.e., surfactant proteins and lamellar bodies) with an average cell thickness of ~4.2 µm (~8 times the cell thickness estimated for RAECM-I) [35] These morphological traits of RAECM-I vs. RAECM-II contribute to the estimated numbers of small (but not large) equivalent pores, which are ~8 times greater in RAECM-I than in RAECM-II. Furthermore, the equivalent pore radii for small and large equivalent pores in RAECM-I and RAECM-II are similar, suggesting that thickness of the epithelial cells governs the number of small equivalent pores but not their equivalent pore radius.

Our current measurements of in vitro permeability and heteropore characteristics of RAECM-I and -II can be compared with these properties determined for the mammalian lung alveolar epithelial barrier obtained in our isolated perfused rat lung studies [1,22], although the latter are complicated by the unknown surface area in intact rat lung studies. Despite the need to use estimates of alveolar surface area for intact lungs, heteropore characteristics of RAECM-I and -II are similar to those estimated for isolated perfused rat or dog lungs [1,22,48,49]. Because in vivo alveolar epithelium is comprised of both AT2 and AT1 cells with a ratio of 2:1, overall heteropore characteristics (including equivalent pore number and area, in addition to equivalent pore radii) of in vivo alveolar epithelium might be more reflective of those for RAECM-II.

It has been reported that there are significant differences in claudin expression in RAECM-I and RAECM-II which may lead to differences in paracellular permeability [50]. Furthermore, decreased expression of claudin 18 in mouse AEC monolayers decreases transepithelial electrical resistance [51], deletion of claudin 4 does not change ion permeability across lung epithelium [52] and increased expression of claudin 3 decreases transepithelial electrical resistance [53]. It remains unclear how claudins create tight junctional pores leading to variable properties of transepithelial electrical resistance and paracellular permeability [4,6,7,12,13,54]. Mathematical modeling of paracellular permeability is a useful method to explore differences in tight junctional pore characteristics among different cell types, while analysis of the molecular basis for claudin (or occludin or zonula occludens-1) expression effects remains to be explored further.

It has been known that the junctional adhesion molecule (JAM) is one of the major transmembrane proteins which form epithelial tight junctions and requires extracellular Ca^++^ to maintain structure of the tight junctions [13,55,56,57,58,59,60]. The integrity of tight junctions is disrupted when exposed to Ca^++^-free media or media containing the calcium chelator EGTA, resulting in decreased transepithelial electrical resistance correlated with formation of tight junctions. Several studies demonstrated that EGTA-induced Ca^++^ depletion from culture media significantly decreases (i.e., >90%) transepithelial electrical resistance of various epithelial cell monolayers, including A6 [57], MDCK [55,56,61], Caco-2 [11] and T84 [11] cells. Consistent with findings reported in the earlier studies, when RAECM-I or -II were treated with 2 mM EGTA for 3 h, transepithelial electrical resistance (i.e., *R_t_*) decreased by ~90% or ~95%, respectively. In addition, under these conditions, tight junction integrity in RAECM-II appeared to be more sensitive to Ca^++^ depletion compared to those in RAECM-I, leading to a higher increase in *P_app_* of hydrophilic solutes. Ca^++^ depletion leads to increased *P_app_* of large molecular weight solutes (e.g., from glycine to 10 kDa dextran) across RAECM-I and -II by up to ~6 and ~400 times, respectively. It has been reported that EGTA-induced tight junctional perturbation dramatically increases paracellular permeability in T84 and Caco-2 cell monolayers, resulting in a loss of size discrimination (or heteropore characteristics) at the tight junctions [11,38].

However, 2 mM EGTA-treated RAECM-I or -II in this study still exhibited heteropore (i.e., small and large equivalent pores) characteristics and restricted paracellular diffusion in a size-selective manner, indicating that Ca^++^ depletion did not induce severe disruption of tight junction integrity but rather dilated small and large equivalent pores of RAECM-I or -II. Our results suggest that Ca^++^ depletion affects primarily the large equivalent pore population in RAECM-I or -II, leading to 1.58 and 4.34 times the equivalent large equivalent pore radii, respectively. With EGTA treatment, existing small equivalent pores in RAECM-I or -II appeared to fuse to form slightly larger equivalent pores, leading to a concomitant decrease in the number of small equivalent pores. This is probably because Ca^++^ depletion induces cellular redistribution of a Ca^++^-dependent adhesion molecule within tight junctions [11,55]. This phenomenon was confirmed by Martinez-Palomo et al. [61] and Meldolesi et al. [62], where Ca^++^ dependent disassembly and reassembly of tight (or occluding) junctions of epithelial cells were visualized by quantitative freeze-fracture electron microscopy. Although further studies are needed to investigate the differential effects of EGTA on *P_app_* of hydrophilic solutes (particularly large molecular weight solutes) or transepithelial electrical resistance, Ca^++^-dependent tight junctional structures of RAECM-I and -II appear to be differentially integrated and/or regulated. Of note is the fact that under severe lung alveolar epithelial injury, passive permeability across the air-blood barrier of the lung may be consistent with our observations for RAECM-I and -II following EGTA treatment. Changes resulting from EGTA treatment indicate that cell–cell interactions are disrupted. Our data suggest that while putative physical pores may be enlarged, the overall two equivalent pore characteristics remain. The changes in equivalent pore sizes are consistent with changes in pore areas. That EGTA could cause equivalent small pores to convert to equivalent large pores is certainly possible conceptually, but there are no experimental data to support that supposition either way. It can be pointed out here that the estimates of the number of pores for experiments with EGTA are associated with considerable uncertainty because the two phases of the *P_app_* versus solute radius response are not as distinctly separated following EGTA as they are without EGTA. The overall model, however, adequately describes both phases of the *P_app_* versus solute radii results as shown in Figure 1 and Figure 2. Using a simpler single equivalent pore model, which would produce a monophasic *P_app_* versus pore size prediction, would in contrast yield a poorer overall fit to the observed data [not shown]. We note that future experimental designs that include additional solutes with radii at both the low and high ends of the two radii ranges demarcated by the reference solute could improve reliability in the estimates for the number of small and large equivalent pores.

Water-filled pores are thought to be part of the tight junctional complexes in epithelia, involving various claudin isoforms, through which permselective passage of ions can take place. Whether or not the same water-filled pores allow passage of hydrophilic solutes is not yet clear. Other water-filled pores are likely organized by tricellulins, occludins, JAMs and other as yet unknown tight junctional proteins, which may represent the routes for hydrophilic solute permeation.

Equivalent pore analyses utilizing transgenic animals involving knockdown/knockout of various claudin isoform(s) can shed light on the role of claudin-type pores. We have shown that claudin 18 knockout mice have a significantly increased permeability to bovine serum albumin compared to control mice (0.56 vs. 0.19) [51], while claudin 4 knockout mice did not show altered permeability to 40 kDa dextran or albumin [63]. It has been suggested that, as intercellular strands seen on freeze fracture increase in number, transepithelial resistance (TER) increases logarithmically [64], a correlation confirmed in some tissues but with many exceptions [65,66,67]. For example, tight MDCK I cell monolayers show much higher TER than leaky MDCK II cell monolayers by 30–60-fold with no significant differences in morphology or the number of TJ strands [60]. Although freeze-fracture studies have been performed utilizing pulmonary tissues [58,68,69], no definitive data on tight junctional strand numbers or physical gap/pore sizes can be found in the literature. In particular, it is known that claudins can form pores of ~0.4 nm diameter in tight junctions of various epithelia [10,70,71]. These claudin-formed pores are believed to be where passage of ions takes place in a cation- or anion-selective manner. Pores formed by claudin 2 or claudin 15 [72,73,74] may allow water passage. It is also known that the tricellular region of epithelia may contain pores of ~10 nm diameter, comprised of tricellulins and other tight junctional proteins (e.g., anguilin 1, 2, and 3) [75,76,77]. Pores formed by tricellulins allow hydrophilic solutes of up to 10 kDa to traverse the MDCK-II cell monolayer, whereas 20 kDa dextrans show much lower *P_app_* and 70 kDa dextrans were excluded altogether from entry into such pores [76]. Because the size of 4, 10, 20 and 70 kDa dextrans is about 3, 5, 7 and 15 nm in diameter, respectively, tricellulin-formed pores of ~10 nm diameter/width are consistent with the total exclusion of 70 kDa dextrans and size-dependent *P_app_* for 4 to 20 kDa dextrans. While it is tempting to associate these physical pores whose diameters are ~0.4 nm (claudins) and ~10 nm (tricellulins) reported in MDCK cells with the equivalent small and large pores in alveolar epithelial cells described in our studies, little published evidence exists that would allow us to confidently make any such conclusions at this time.

In summary, both RAECM-I and -II exhibit tight junctional equivalent water-filled cylindrical pores with a radius of 0.32~0.41 nm, with these small equivalent pores occupying >99% of the total equivalent pore area. The number of these small equivalent pores in RAECM-II is similar to that in RAECM-I. The large equivalent pore size in RAECM-I and -II is 11.56 nm and 9.88 nm, respectively. Large equivalent pores occupy 0.03% and 0.08% of total equivalent pore area in RAECM-I and -II, respectively. The number of large equivalent pores in RAECM-II is similar to that in RAECM-I. In addition, perturbation of tight junction assembly by Ca^++^ depletion leads to increased small and large equivalent pore sizes for both RAECM-I and -II. EGTA treatment of RAECM increased the equivalent pore size but decreased the number of small equivalent pores. As for large equivalent pores in RAECM-I and -II, EGTA treatment led to a dramatic increase in equivalent pore size, with moderate increase in the number of large equivalent pores. Little information is available on the relationship between the molecular basis for regulation and physiological function of tight junctions (and their components) in alveolar epithelial type I or type II cells. Modeling of paracellular permeability presented here may help lead to improved understanding of physiological characteristics of tight junctions in the alveolar epithelium.

## Figures and Tables

**Table 1:** Physicochemical properties of hydrophilic solutes.

Solute	*Mw* (Daltons)	*ρ* (g/cm^3^)	*D* (cm^2^/s × 10^−5^) at 37 °C	*r_e_* (nm)	*r_0_* (nm)	*r* (nm)
Formamide (1-^14^C)	45.04	1.133	2.20 ^(a)^	0.14 ^(h)^	0.25 ^(j)^	0.19 ^(k)^
Acetamide (1-^14^C)	59.07	1.160	1.75 ^(a)^	0.17 ^(h)^	0.27 ^(j)^	0.22 ^(k)^
Ethylene glycol (1,2-^14^C)	62.1	1.113	1.59 ^(b)^	0.19 ^(h)^	0.28 ^(j)^	0.23 ^(k)^
Glycine (1-^14^C)	75.1	1.161	1.38 ^(c)^	0.22 ^(h)^	0.29 ^(j)^	0.25 ^(k)^
D-Arabinose (1-^14^C)	150.1	1.625	1.06 ^(d)^	0.29 ^(h)^	0.33 ^(j)^	0.31 ^(k)^
D-Mannitol (1-^14^C)	182.2	1.520	0.91 ^(e)^	0.35 ^(h)^	0.36 ^(j)^	0.36 ^(k)^
Sucrose (^14^C(U))	342.3	1.587	0.72 ^(d)^	0.43 ^(h)^	0.44 ^(j)^	0.44 ^(k)^
5-Carboxyfluorescein	376.3	-	0.64 ^(f)^	0.49 ^(h)^	-	0.49
Sulforhodamine B	558.7	-	0.46 ^(f)^	0.68 ^(h)^	-	0.68
FITC-4 kDa dextran	~4457	-	0.157 ^(g)^	1.61 ^(i)^	-	1.61
FITC-10 kDa dextran	~9479	-	0.106 ^(g)^	2.29 ^(i)^	-	2.29

*D* was obtained from (a) Gary-Bobo and Weber [27], (b) Byers and King [28], (c) Longsworth [29], (d) Mogi et al. [30], (e) Peck et al. [31], (f) the Wilke-Chang equation [32] *D* = [ 7.4 × 10^−8^ × ( 2.6 · *M* )^1/2^ · *T*]/[*η* · *V*^0.6^], where *M* is molecular weight of water, *T* is absolute temperature (300 K), *η* is viscosity of water (0.7 mPa·s) and *V* is molecular volume and (g) Deen et al. [33]; *r_e_* was calculated from (h) the Stokes–Einstein equation *r_e_* = ( *k* · *T* )*/*( 6 · *π* · *η* · *D* ), where *k* is the Boltzmann constant (1.38 × 10^−23^ J/K); (i) *r_e_* = 0.33 × ( *Mw* )^0.463^ [26]; (j) *r_0_* was calculated from *r_0_* = ( 3 · *Mw* )*/*( 4 · *π* · *ρ* · *N )*^1/3^, where *ρ* is density and *N* is Avogadro’s number (6.02214 × 10^23^) [25]; and (k) *r* was calculated as ( *r_e_* + *r_0_* )/2.

**Table 2:** Apparent permeability (*P_app_*) of benzyl alcohol and unstirred layer thickness (*δ*) of RAECM-I and -II.

RAECM-I		RAECM-II	
*P_app_* (cm/s) × 10^−5^	*δ* (cm)	*P_app_* (cm/s) × 10^−5^	*δ* (cm)
6.51 ± 0.67	0.184 ± 0.017	6.09 ± 1.03	0.199 ± 0.036

Comparison of the benzyl alcohol *P_app_* across RAECM-I and that across RAECM-II or comparison of unstirred layer thickness of RAECM-I and that of RAECM-II shows no statistical difference.

**Table 3:** *P_app_* across RAECM-I and RAECM-II with statistical analyses. (**a**) *P_app_* across RAECM-I and RAECM-II in the absence and presence of EGTA corrected for unstirred layer effects. (**b**) and (**c**) Three-way analysis of variances (ANOVA) of *P_app_* across RAECM-I and -II (with or without EGTA). Three factors assigned for three-way ANOVA are as follows: “different solutes”, “wo vs. w EGTA”, and “RACEM-I vs. RAECM-II” with alpha being set at 0.05, where w = with, wo = without, × = interaction between factors, and DF = degree of freedom. Results suggest that there are significant differences between *P_app_* across RAECM-I and those across RAECM-II, significant effects of EGTA treatment on *P_app_* for both RAECM-I and RAECM-II, and *P_app_* for different solutes in each experimental setting are statistically significant (all at *p* < 0.0001). (**c**) Summary table of three-way ANOVA for the source of variation, % of variation, *p* value, *p* value summary, and significance vs. no significance. SS (sum of square), DF (degree of freedom), MS (mean square).

(**a**)												
**Solutes**	***P_app_* (cm/s) × 10^−7^**											
	**RAECM-I**						**RAECM-II**					
	**Untreated**			**EGTA Treated**			**Untreated**			**EGTA Treated**		
	**Mean**	**SD**	**n**	**Mean**	**SD**	**n**	**Mean**	**SD**	**n**	**Mean**	**SD**	**n**
Formamide	55.039	4.094	6	92.761	12.368	3	68.455	11.572	8	337.655	218.957	6
Acetamide	91.555	12.191	8	110.919	5.068	6	105.353	11.416	9	387.064	92.762	3
Ethylene glycol	58.673	4.131	9	74.994	29.331	6	74.961	7.031	9	153.386	32.461	6
Glycine	4.011	0.269	7	24.168	2.581	4	7.184	0.920	8	358.107	79.375	2
Arabinose	2.641	1.012	9	15.634	4.113	6	1.322	0.398	9	95.249	50.396	6
Mannitol	2.086	0.256	6	23.468	2.542	6	2.297	0.475	9	85.370	17.206	5
Sucrose	0.869	0.141	6	11.156	2.341	5	0.676	0.261	9	37.870	2.705	3
5-Carboxyfluorescein	0.644	0.222	8	6.120	0.361	3	0.556	0.196	6	37.754	4.499	5
Sulforhodamine B	0.466	0.083	6	6.088	0.447	3	0.453	0.200	6	37.672	9.336	5
FITC-4 kDa dextran	0.180	0.067	9	2.541	0.112	3	0.120	0.019	6	9.228	1.707	3
FITC-10 kDa dextran	0.057	0.020	8	1.142	0.130	3	0.026	0.005	9	10.183	1.709	3
(**b**)												
**Source of Variation**							**% of Total Variation**				***p* Value**	
different solutes							41.250				<0.0001	
wo vs. w EGTA							13.190				<0.0001	
RAECM-I vs. RAECM-II							9.550				<0.0001	
different solutes × wo vs. w EGTA							10.820				<0.0001	
different solutes × RAECM-I vs. RAECM-II							9.542				<0.0001	
wo vs. w EGTA × RAECM-I vs. RAECM-II							8.193				<0.0001	
different solutes × wo vs. w EGTA × RAECM-I vs. RAECM-II							8.303				<0.0001	
(**c**)												
**ANOVA Table**	**SS (Type III)**			**DF**	**MS**			**F (DFn, DFd)**			***p* Value**	
different solutes	7.52 × 10^−9^			10	7.52 × 10^−10^			F (10, 221) = 56.96			*p* < 0.0001	
wo vs. w EGTA	2.41 × 10^−9^			1	2.41 × 10^−9^			F (1, 221) = 182.20			*p* < 0.0001	
RAECM-I vs. RAECM-II	1.74 × 10^−9^			1	1.74 × 10^−9^			F (1, 221) = 131.90			*p* < 0.0001	
different solutes × wo vs. w EGTA	1.97 × 10^−9^			10	1.97 × 10^−9^			F (10, 221) = 14.94			*p* < 0.0001	
different solutes × RAECM-I vs. RAECM-II	1.74 × 10^−9^			10	1.74 × 10^−10^			F (10, 221) = 13.18			*p* < 0.0001	
wo vs. w EGTA × RAECM-I vs. RAECM-II	1.49 × 10^−9^			1	1.49 × 10^−9^			F (1, 221) = 113.10			*p* < 0.0001	
different solutes × wo vs. w EGTA × RAECM-I vs. RAECM-II	1.51 × 10^−9^			10	1.51 × 10^−10^			F (10, 221) = 11.47			*p* < 0.0001	
Residual	2.92 × 10^−9^			221	1.32 × 10^−11^							

**Table 4:** Heteropore characteristics of RAECM-I. Number and radius of small and large equivalent pore populations based on maximum likelihood optimization approach using all entries of *P_app_* in the absence and presence of EGTA. Equivalent pore number is per nominal surface area (1 cm^2^) of RAECM-I.

RAECM-I		Estimated Pore Radius (nm)	Standard Error (%)	Estimated Number of Pores	Standard Error (%)
**without EGTA**	Small pores	0.32	1.7	9.15 × 10^11^	20.6
Cf. Reference solute radius = 0.35 nm	Large pores	11.56	16.7	1.88 × 10^5^	41.9
**with EGTA**	Small pores	0.59	30.3	1.14 × 10^10^	148.2
Cf. Reference solute radius = 0.30 nm	Large pores	18.3	33.2	8.92 × 10^5^	72.9

**Table 5:** Small and large equivalent pore areas and their fractions and fold changes in equivalent pore area in response to EGTA treatment of RAECM-I. Equivalent pore characteristics in response to EGTA treatment. Entries are based on the estimated values listed in Table 4. Equivalent pore area is per nominal surface area (1 cm^2^) of RAECM-I.

RAECM-I	Pore Area (cm^2^)		% of Total Pore Area		Fold Change in Pore Area	
	Small	Large	Small	Large	Small	Large
without EGTA	3.03 × 10^−3^	7.90 × 10^−7^	99.97	0.03	1.0	1.0
with EGTA	1.23 × 10^−4^	9.38 × 10^−6^	92.91	7.09	0.9	271.9

**Table 6:** Heteropore characteristics of RAECM-II. Radius and number of small and large equivalent pore populations based on maximum likelihood optimization approach using all entries of *P_app_* in the absence and presence of EGTA. Equivalent pore number is per nominal surface area (1 cm^2^) of RAECM-II.

RAECM-II		Estimated Pore Radius (nm)	Standard Error (%)	Estimated Number of pores	Standard Error (%)
without EGTA	Small pores	0.41	4.0	1.07 × 10^11^	34.0
Cf. Reference solute radius = 0.40 nm	Large pores	9.88	14.4	1.45 × 10^5^	38.2
with EGTA	Small pores	0.78	25.1	7.85 × 10^10^	94.9
Cf. Reference solute radius = 0.45 nm	Large pores	42.89	123.6	5.43 × 10^6^	261.0

**Table 7:** Small and large equivalent pore areas and their fractions and fold changes in equivalent pore area in response to EGTA treatment of RAECM-II. Entries are based on the estimated values listed in Table 6. Equivalent pore area is per nominal surface area (1 cm^2^) of RAECM-II.

RAECM-II	Pore Area (cm^2^)		% of Total Pore Area		Fold Change in Pore Area	
	Small	Large	Small	Large	Small	Large
without EGTA	5.588 × 10^−4^	4.445 × 10^−7^	99.92	0.08	1.0	1.0
with EGTA	1.500 × 10^−3^	3.135 × 10^−4^	82.71	17.29	0.8	217.5

## Appendix A

**Table A1:** Comparison of various *P_app_* across RAECM-I (**a**) and RAECM-II (**b**), RAECM-I treated with EGTA (**c**), and RAECM-II treated with EGTA (**d**). Solute permeation via small pores (as shown in Figure 1 and Figure 2) is in general much greater than that via large pores across both RAECM-I and RAECM-II irrespective of EGTA treatmen. Comparisons with adjusted *p* values < 0.05 are listed. **** denotes *p* < 0.0001, *** denotes *p* < 0.001, ** denotes *p* < 0.01 and * denotes *p* < 0.05.

(**a**) Comparison table for *P_app_* for RAECM-I.											
***P_app_* of RAECM-I**	***P_app_* of RAECM-I**										
	**Formamide**	**Acetamide**	**Ethylene Glycol**	**Glycine**	**Arabinose**	**Mannitol**	**Sucrose**	**5-Carboxy- Fluorescein**	**Sulfo- Rhodamine B**	**FITC-4 kDa Dextran**	**FITC-10 kDa Dextran**
Formamide											
Acetamide				**	***	**	**	***	**	***	***
Ethylene glycol											
Glycine											
Arabinose											
Mannitol											
Sucrose											
5-Carboxyfluorescein											
Sulforhodamine B											
FITC-4 kDa dextran											
FITC-10 kDa dextran											
(**b**) Comparison table for *P_app_* for RAECM-II.											
***P_app_* of RAECM-II**	***P_app_* of RAECM-II**										
	**Formamide**	**Acetamide**	**Ethylene glycol**	**Glycine**	**Arabinose**	**Mannitol**	**Sucrose**	**5-Carboxy- Fluorescein**	**Sulfo- Rhodamine B**	**FITC-4 kDa Dextran**	**FITC-10 kDa Dextran**
Formamide											
Acetamide				****	****	****	****	***	***	****	****
Ethylene glycol					*	*	*			*	*
Glycine											
Arabinose											
Mannitol											
Sucrose											
5-Carboxyfluorescein											
Sulforhodamine B											
FITC-4 kDa dextran											
FITC-10 kDa dextran											
(**c**) Comparison table for *P_app_* for RAECM-I with EGTA.											
***P_app_* of RAECM-I with EGTA**	***P_app_* of RAECM-I with EGTA**										
	**Formamide**	**Acetamide**	**Ethylene glycol**	**Glycine**	**Arabinose**	**Mannitol**	**Sucrose**	**5-Carboxy- Fluorescein**	**Sulfo- Rhodamine B**	**FITC-4 kDa Dextran**	**FITC-10 kDa Dextran**
Formamide											
Acetamide					**	*	**	*	*	*	*
Ethylene glycol											
Glycine											
Arabinose											
Mannitol											
Sucrose											
5-Carboxyfluorescein											
Sulforhodamine B											
FITC-4 kDa dextran											
FITC-10 kDa dextran											
(**d**) Comparison table for *P_app_* for RAECM-II with EGTA.											
**Papp of RAECM-II with EGTA**	**Papp of RAECM-II with EGTA**										
	**Formamide**	**Acetamide**	**Ethylene glycol**	**Glycine**	**Arabinose**	**Mannitol**	**Sucrose**	**5-Carboxy- Fluorescein**	**Sulfo- Rhodamine B**	**FITC-4 kDa Dextran**	**FITC-10 kDa Dextran**
Formamide			****		****	****	****	****	****	****	****
Acetamide			****		****	****	****	****	****	****	****
Ethylene glycol				****			**	***	***	****	****
Glycine					****	****	****	****	****	****	****
Arabinose											
Mannitol											
Sucrose											
5-Carboxyfluorescein											
Sulforhodamine B											
FITC-4 kDa dextran											
FITC-10 kDa dextran											

**Table A2:** Comparisons for *P_app_* of RAECM-I vs -II with or without EGTA based on three-way ANOVA and post-hoc Tukey’s multiple comparisons. No entry denotes no significance between the two *P_app_*. Comparisons with adjusted *p* values < 0.05 are listed. **** denotes *p* < 0.0001, *** denotes *p* < 0.001, ** denotes *p* < 0.01 and * denotes *p* < 0.05.

(**a**) Comparison table for *P_app_* of RAECM-I vs RAECM-II.											
***P_app_* of RAECM-I**	***P_app_* of RAECM-II**										
	**Formamide**	**Acetamide**	**Ethylene glycol**	**Glycine**	**Arabinose**	**Mannitol**	**Sucrose**	**5-Carboxy- Fluorescein**	**Sulfo- Rhodamine B**	**FITC-4 kDa Dextran**	**FITC-10 kDa Dextran**
Formamide											
Acetamide				**	***	***	***	**	**	**	***
Ethylene glycol											
Glycine		****									
Arabinose		****	*								
Mannitol		***									
Sucrose		***									
5-Carboxyfluorescein		****	*								
Sulforhodamine B		***									
FITC-4 kDa dextran		****									
FITC-10 kDa dextran		****	*								
(**b**) Comparison table for *P_app_* of RAECM-I vs RAECM-I with EGTA.											
***P_app_* of RAECM-I**	***P_app_* of RAECM-I with EGTA**										
	**Formamide**	**Acetamide**	**Ethylene glycol**	**Glycine**	**Arabinose**	**Mannitol**	**Sucrose**	**5-Carboxy- Fluorescein**	**Sulfo- Rhodamine B**	**FITC-4 kDa Dextran**	**FITC-10 kDa Dextran**
Formamide											
Acetamide											
Ethylene glycol											
Glycine		***									
Arabinose		****									
Mannitol		***									
Sucrose		***									
5-Carboxyfluorescein		****									
Sulforhodamine B		***									
FITC-4 kDa dextran		****									
FITC-10 kDa dextran		****									
(**c**) Comparison table for *P_app_* of RAECM-I vs RAECM-II with EGTA.											
***P_app_* of RAECM-I**	***P_app_* of RAECM-II with EGTA**										
	**Formamide**	**Acetamide**	**Ethylene glycol**	**Glycine**	**Arabinose**	**Mannitol**	**Sucrose**	**5-Carboxy- Fluorescein**	**Sulfo- Rhodamine B**	**FITC-4 kDa Dextran**	**FITC-10 kDa Dextran**
Formamide	****	****	**	****				****			
Acetamide	****	****		****							
Ethylene glycol	****	****	**	****							
Glycine	****	****	****	****	**						
Arabinose	****	****	****		**	*					
Mannitol	****	****	****	****	**						
Sucrose	****	****	****	****	**						
5-Carboxyfluorescein		****	****	****	**	*					
Sulforhodamine B	****	****	****	****	**						
FITC-4 kDa dextran	****	****	****	****	**	*					
FITC-10 kDa dextran	****	****	****	****	**	*					
(**d**) Comparison table for *P_app_* of RAECM-II vs RAECM-I with EGTA.											
***P_app_* of RAECM-II**	***P_app_* of RAECM-I with EGTA**										
	**Formamide**	**Acetamide**	**Ethylene glycol**	**Glycine**	**Arabinose**	**Mannitol**	**Sucrose**	**5-Carboxy- Fluorescein**	**Sulfo- Rhodamine B**	**FITC-4 kDa Dextran**	**FITC-10 kDa Dextran**
Formamide											
Acetamide					**	*	**	*	*	*	*
Ethylene glycol											
Glycine		***									
Arabinose		****									
Mannitol		****									
Sucrose		****									
5-Carboxyfluorescein		***									
Sulforhodamine B		***									
FITC-4 kDa dextran		***									
FITC-10 kDa dextran		****									
(**e**) Comparison table for *P_app_* of RAECM-II vs RAECM-II with EGTA.											
***P_app_* of RAECM-II**	***P_app_* of RAECM-II with EGTA**										
	**Formamide**	**Acetamide**	**Ethylene glycol**	**Glycine**	**Arabinose**	**Mannitol**	**Sucrose**	**5-Carboxy- Fluorescein**	**Sulfo- Rhodamine B**	**FITC-4 kDa Dextran**	**FITC-10 kDa Dextran**
Formamide	****	****	*	****				****	****		
Acetamide	****	****		****							
Ethylene glycol	****	****	*	****							
Glycine	****	****	****	****	**						
Arabinose	****	****	****		**	*					
Mannitol	****	****	****	****	**	*					
Sucrose		****	****	****	**	*					
5-Carboxyfluorescein		****	****	****	**						
Sulforhodamine B		****	****	****	**						
FITC-4 kDa dextran	****	****	****	****	**						
FITC-10 kDa dextran	****	****	****	****	**	*					
(**f**) Comparison table for *P_app_* of RAECM-I with EGTA vs RAECM-II with EGTA.											
***P_app_* of RAECM-I** **with EGTA**	***P_app_* of RAECM-II with EGTA**										
	**Formamide**	**Acetamide**	**Ethylene Glycol**	**Glycine**	**Arabinose**	**Mannitol**	**Sucrose**	**5-Carboxy- Fluorescein**	**Sulfo- Rhodamine B**	**FITC-4 kDa Dextran**	**FITC-10 kDa Dextran**
Formamide	****	****		****							
Acetamide	****	****		****							
Ethylene glycol	****	****		****							
Glycine	****	****	****	****							
Arabinose	****	****	****	****							
Mannitol	****	****	****	****							
Sucrose	****	****	****	****							
5-Carboxyfluorescein	****	****	****	****							
Sulforhodamine B	****	****	****	****							
FITC-4 kDa dextran	****	****	****	****							
FITC-10 kDa dextran	****	****	****	****							

**Table A3:** Number and radius of small and large equivalent pore populations based on maximum likelihood optimization approach using the mean of *P_app_* for each solute in the absence and presence of EGTA. Equivalent pore number is per nominal surface area (1 cm^2^) of RAECM-I. Standard errors are much larger than those obtained using all entries of *P_app_*.

RAECM-I		Estimated Pore Radius (nm)	Standard Error (%)	Estimated Number of Pores	Standard Error (%)
without EGTA	Small pores	0.32	3.7	1.01 × 10^12^	46.9
Cf. Reference solute radius = 0.35 nm	Large pores	10.02	35.2	2.63 × 10^5^	90.1
with EGTA	Small pores	0.42	25.7	7.01 × 10^10^	177.2
Cf. Reference solute radius = 0.30 nm	Large pores	21.61	73.5	5.98 × 10^5^	162.3

**Table A4:** Equivalent pore characteristics in response to EGTA treatment. Entries are based on the estimated values listed in Table A3. Equivalent pore area is per nominal surface area (1 cm^2^) of RAECM-I.

RAECM-I	Pore Area (cm^2^)		% of Total Pore Area		Fold Change in Pore Area	
	Small	Large	Small	Large	Small	Large
without EGTA	3.27 × 10^−3^	8.28 × 10^7^	99.97	0.03	1.0	1.0
with EGTA	2.34 × 10^−5^	8.77 × 10^−6^	72.76	27.24	0.7	1044.2

**Table A5:** Radius and number of small and large equivalent pore populations based on maximum likelihood optimization approach using the mean of *P_app_* for each solute in the absence and presence of EGTA. Equivalent pore number is per nominal surface area (1 cm^2^) of RAECM-II.

RAECM-II		Estimated Pore Radius (nm)	Standard Error (%)	Estimated Number of Pores	Standard Error (%)
without EGTA	Small pores	0.41	2.7	1.42 × 10^12^	22.9
Cf. Reference solute radius = 0.40 nm	Large pores	8.83	8.1	2.34 × 10^6^	22.0
with EGTA	Small pores	0.92	72.7	5.33 × 10^10^	257.7
Cf. Reference solute radius = 0.35 nm	Large pores	39.34	147.6	7.07 × 10^6^	311.5

**Table A6:** B. Equivalent pore characteristics in response to EGTA treatment. Entries are based on the estimated values listed in Table A5. Equivalent pore area is per nominal surface area (1 cm^2^) of RAECM-II.

RAECM-II	Pore Area (cm^2^)		% of Total Pore Area		Fold Change in Pore Area	
	Small	Large	Small	Large	Small	Large
without EGTA	7.346 × 10^−3^	5.730 × 10^−6^	99.92	0.08	1.0	1.0
with EGTA	1.411 × 10^−3^	3.437 × 10^−4^	80.42	19.58	0.8	246.4

## References

[1] Berg M.M., Kim K.J., Lubman R.L., Crandall E.D. (1989). Hydrophilic solute transport across rat alveolar epithelium. J. Appl. Physiol..

[2] Crandall E.D., Staub N.C., Goldberg H.S., Effros R.M. (1983). Recent developments in pulmonary edema. Ann. Intern. Med..

[3] Koval M. (2009). Tight junctions, but not too tight: Fine control of lung permeability by claudins. Am. J. Physiol. Lung Cell Mol. Physiol..

[4] Schlingmann B., Molina S.A., Koval M. (2015). Claudins: Gatekeepers of lung epithelial function. Semin. Cell Dev. Biol..

[5] Wittekindt O.H. (2017). Tight junctions in pulmonary epithelia during lung inflammation. Pflugers Arch..

[6] Van Itallie C.M., Anderson J.M. (2004). The molecular physiology of tight junction pores. Physiology.

[7] Anderson J.M. (2001). Molecular structure of tight junctions and their role in epithelial transport. Physiology.

[8] Kim K.J., Crandall E.D. (1983). Heteropore populations of bullfrog alveolar epithelium. J. Appl. Physiol. Respir. Environ. Exerc. Physiol..

[9] Linnankoski J., Makela J., Palmgren J., Mauriala T., Vedin C., Ungell A.L., Lazorova L., Artursson P., Urtti A., Yliperttula M. (2010). Paracellular porosity and pore size of the human intestinal epithelium in tissue and cell culture models. J. Pharm. Sci..

[10] Van Itallie C.M., Holmes J., Bridges A., Gookin J.L., Coccaro M.R., Proctor W., Colegio O.R., Anderson J.M. (2008). The density of small tight junction pores varies among cell types and is increased by expression of claudin-2. J. Cell Sci..

[11] Watson C.J., Rowland M., Warhurst G. (2001). Functional modeling of tight junctions in intestinal cell monolayers using polyethylene glycol oligomers. Am. J. Physiol. Cell Physiol..

[12] Gunzel D., Yu A.S. (2013). Claudins and the modulation of tight junction permeability. Physiol. Rev..

[13] Tsukita S., Furuse M., Itoh M. (2001). Multifunctional strands in tight junctions. Nat. Rev. Mol. Cell Biol.

[14] Kim K.J., Borok Z., Crandall E.D. (2001). A useful in vitro model for transport studies of alveolar epithelial barrier. Pharm. Res..

[15] Cheek J.M., Evans M.J., Crandall E.D. (1989). Type I cell-like morphology in tight alveolar epithelial monolayers. Exp. Cell Res..

[16] Borok Z., Danto S.I., Zabski S.M., Crandall E.D. (1994). Defined medium for primary culture de novo of adult rat alveolar epithelial cells. In Vitro Cell Dev. Biol. Anim..

[17] Borok Z., Hami A., Danto S.I., Zabski S.M., Crandall E.D. (1995). Rat serum inhibits progression of alveolar epithelial cells toward the type I cell phenotype in vitro. Am. J. Respir. Cell Mol. Biol..

[18] Borok Z., Lubman R.L., Danto S.I., Zhang X.L., Zabski S.M., King L.S., Lee D.M., Agre P., Crandall E.D. (1998). Keratinocyte growth factor modulates alveolar epithelial cell phenotype in vitro: Expression of aquaporin 5. Am. J. Respir. Cell Mol. Biol..

[19] Borok Z., Mihyu S., Fernandes V.F., Zhang X.L., Kim K.J., Lubman R.L. (1999). KGF prevents hyperoxia-induced reduction of active ion transport in alveolar epithelial cells. Am. J. Physiol..

[20] Qiao R., Yan W., Clavijo C., Mehrian-Shai R., Zhong Q., Kim K.J., Ann D., Crandall E.D., Borok Z. (2008). Effects of KGF on alveolar epithelial cell transdifferentiation are mediated by JNK signaling. Am. J. Respir. Cell Mol. Biol..

[21] Crandall E.D., Kim K.J. (1981). Transport of water and solutes across bullfrog alveolar epithelium. J. Appl. Physiol. Respir. Environ. Exerc. Physiol..

[22] McLaughlin G.E., Kim K.J., Berg M.M., Agoris P., Lubman R.L., Crandall E.D. (1993). Measurement of solute fluxes in isolated rat lungs. Respir. Physiol..

[23] Garner F.H., Marchant P.J.M. (1961). Diffusivities of associated compounds in water. Trans. Instn. Chem. Engrs..

[24] Pietras R.J., Wright E.M. (1975). The membrane action of antidiuretic hormone (ADH) on toad urinary bladder. J. Membr. Biol..

[25] Renkin E.M. (1954). Filtration, diffusion, and molecular sieving through porous cellulose membranes. J. Gen. Physiol..

[26] Venturoli D., Rippe B. (2005). Ficoll and dextran vs. globular proteins as probes for testing glomerular permselectivity: Effects of molecular size, shape, charge, and deformability. Am. J. Physiol. Ren. Physiol..

[27] Gary-Bobo C., Weber H.W. (1969). Diffusion of alcohols and amides in water from 4 to 37°. J. Phys. Chem..

[28] Byers C.H., King C.J. (1966). Liquid diffusivities in the glycol-water system. J. Phys. Chem..

[29] Longsworth L.G. (1953). Diffusion measurements, at 25°, of aqueous solutions of amino acids, peptides and sugars. J. Am. Chem. Soc..

[30] Mogi N., Sugai E., Fuse Y., Funazukuri T. (2007). Infinite dilution binary diffusion coefficients for six sugars at 0.1 MPa and temperatures from (273.2 to 353.2) K. J. Chem Eng. Data.

[31] Peck K.D., Ghanem A.H., Higuchi W.I. (1994). Hindered diffusion of polar molecules through and effective pore radii estimates of intact and ethanol treated human epidermal membrane. Pharm. Res..

[32] Wilke C.R., Chang P. (1955). Correlation of diffusion coefficients in dilute solutions. AIChE J..

[33] Deen W.M., Bohrer M.P., Epstein N.B. (1981). Effects of molecular size and configuration on diffusion in microporous membranes. AIChE J..

[34] Levitt D.G. (1975). General continuum analysis of transport through pores. I. Proof of Onsager’s reciprocity postulate for uniform pore. Biophys. J..

[35] Borok Z., Danto S.I., Dimen L.L., Zhang X.L., Lubman R.L. (1998). Na^+^-K^+^-ATPase expression in alveolar epithelial cells: Upregulation of active ion transport by KGF. Am. J. Physiol..

[36] D’Argenio D.Z., Schumitzky A., Wang X. (2009). ADAPT 5 User’s Guide: Pharmacokinetic/Pharmacodynamic Systems Analysis Software.

[37] Adson A., Raub T.J., Burton P.S., Barsuhn C.L., Hilgers A.R., Audus K.L., Ho N.F. (1994). Quantitative approaches to delineate paracellular diffusion in cultured epithelial cell monolayers. J. Pharm. Sci..

[38] Knipp G.T., Ho N.F., Barsuhn C.L., Borchardt R.T. (1997). Paracellular diffusion in Caco-2 cell monolayers: Effect of perturbation on the transport of hydrophilic compounds that vary in charge and size. J. Pharm. Sci..

[39] Cavanaugh K.J., Cohen T.S., Margulies S.S. (2006). Stretch increases alveolar epithelial permeability to uncharged micromolecules. Am. J. Physiol. Cell Physiol.

[40] Dodoo A.N.O., Bansal S.S., Barlow D.J., Bennet F., Hider R.C., Lansley A.B., Lawrence M.J., Marriott C. (2000). Use of alveolar cell monolayers of varying electrical resistance to measure pulmonary peptide transport. J. Pharm. Sci..

[41] Dodoo A.N., Bansal S., Barlow D.J., Bennet F.C., Hider R.C., Lansley A.B., Lawrence M.J., Marriott C. (2000). Systematic investigations of the influence of molecular structure on the transport of peptides across cultured alveolar cell monolayers. Pharm. Res..

[42] Smyth D.H., Wright E.M. (1966). Streaming potentials in the rat small intestine. J. Physiol..

[43] Whittembury G. (1962). Action of antidiuretic hormone on the equivalent pore radius at both surfaces of the epithelium of the isolated toad skin. J. Gen. Physiol..

[44] Wright E.M., Pietras R.J. (1974). Routes of nonelectrolyte permeation across epithelial membranes. J. Membr. Biol..

[45] Matsukawa Y., Lee V.H., Crandall E.D., Kim K.J. (1997). Size-dependent dextran transport across rat alveolar epithelial cell monolayers. J. Pharm. Sci..

[46] Theodore J., Robin E.D., Gaudio R., Acevedo J. (1975). Transalveolar transport of large polar solutes (sucrose, inulin, and dextran). Am. J. Physiol..

[47] Bahhady R., Kim K.J., Borok Z., Crandall E.D., Shen W.C. (2007). Enhancement of insulin transport across primary rat alveolar epithelial cell monolayers by endogenous cellular factor(s). Pharm. Res..

[48] Conhaim R.L., Eaton A., Staub N.C., Heath T.D. (1988). Equivalent pore estimate for the alveolar-airway barrier in isolated dog lung. J. Appl. Physiol..

[49] Conhaim R.L., Watson K.E., Lai-Fook S.J., Harms B.A. (2001). Transport properties of alveolar epithelium measured by molecular hetastarch absorption in isolated rat lungs. J. Appl. Physiol..

[50] Wang F., Daugherty B., Keise L.L., Wei Z., Foley J.P., Savani R.C., Koval M. (2003). Heterogeneity of claudin expression by alveolar epithelial cells. Am. J. Respir. Cell Mol. Biol..

[51] Li G., Flodby P., Luo J., Kage H., Sipos A., Gao D., Ji Y., Beard L.L., Marconett C.N., DeMaio L. (2014). Knockout mice reveal key roles for claudin 18 in alveolar barrier properties and fluid homeostasis. Am. J. Respir. Cell Mol. Biol..

[52] Kage H., Flodby P., Gao D., Kim Y.H., Marconett C.N., DeMaio L., Kim K.J., Crandall E.D., Borok Z. (2014). Claudin 4 knockout mice: Normal physiological phenotype with increased susceptibility to lung injury. Am. J. Physiol. Lung Cell Mol. Physiol..

[53] Mitchell L.A., Overgaard C.E., Ward C., Margulies S.S., Koval M. (2011). Differential effects of claudin-3 and claudin-4 on alveolar epithelial barrier function. Am. J. Physiol. Lung Cell Mol. Physiol..

[54] Overgaard C.E., Mitchell L.A., Koval M. (2012). Roles for claudins in alveolar epithelial barrier function. Ann. N. Y. Acad. Sci..

[55] Collares-Buzato C.B., McEwan G.T., Jepson M.A., Simmons N.L., Hirst B.H. (1994). Paracellular barrier and junctional protein distribution depend on basolateral extracellular Ca^2+^ in cultured epithelia. Biochim. Biophys. Acta.

[56] Gonzalez-Mariscal L., Contreras R.G., Bolivar J.J., Ponce A., Chavez De Ramirez B., Cereijido M. (1990). Role of calcium in tight junction formation between epithelial cells. Am. J. Physiol..

[57] Jovov B., Lewis S.A., Crowe W.E., Berg J.R., Wills N.K. (1994). Role of intracellular Ca^2+^ in modulation of tight junction resistance in A6 cells. Am. J. Physiol..

[58] Rothen-Rutishauser B., Riesen F.K., Braun A., Gunthert M., Wunderli-Allenspach H. (2002). Dynamics of tight and adherens junctions under EGTA treatment. J. Membr. Biol..

[59] Schneeberger E.E., Lynch R.D. (1992). Structure, function, and regulation of cellular tight junctions. Am. J. Physiol..

[60] Stevenson B.R., Anderson J.M., Goodenough D.A., Mooseker M.S. (1988). Tight junction structure and ZO-1 content are identical in two strains of Madin-Darby canine kidney cells which differ in transepithelial resistance. J. Cell Biol..

[61] Martinez-Palomo A., Meza I., Beaty G., Cereijido M. (1980). Experimental modulation of occluding junctions in a cultured transporting epithelium. J. Cell Biol..

[62] Meldolesi J., Castiglioni G., Parma R., Nassivera N., De Camilli P. (1978). Ca^++^-dependent disassembly and reassembly of occluding junctions in guinea pig pancreatic acinar cells. Effect of drugs. J. Cell Biol..

[63] Wray C., Mao Y., Pan J., Chandrasena A., Piasta F., Frank J.A. (2009). Claudin 4 augments alveolar epithelial barrier function and is induced in acute lung injury. Am. J. Physiol. Lung Cell Mol. Physiol..

[64] Claude P., Goodenough D.A. (1973). Fracture faces of zonulae occludentes from “tight” and “leaky” epithelia. J. Cell Biol..

[65] Frederiksen O., Mollgard K., Rostgaard J. (1979). Lack of correlation between transepithelial transport capacity and paracellular pathway ultrastructure in Alcian blue-treated rabbit gallbladders. J. Cell Biol..

[66] Martinez-Palomo A., Erlij D. (1975). Structure of tight junctions in epithelia with different permeability. Proc. Natl. Acad. Sci. USA.

[67] Mollgard K., Milinowska D.H., Saunders N.R. (1976). Lack of correlation between tight junction morphology and permeability properties in developing choroid plexus. Nature.

[68] Schneeberger-Keeley E.E., Karnovsky M.J. (1968). The ultrastructural basis of alveolar-capillary membrane permeability to peroxidase used as a tracer. J. Cell Biol..

[69] Walker D.C., MacKenzie A.L., Wiggs B.R., Montaner J.G., Hogg J.C. (1988). Assessment of tight junctions between pulmonary epithelial and endothelial cells. J. Appl. Physiol..

[70] Furuse M., Furuse K., Sasaki H., Tsukita S. (2001). Conversion of zonulae occludentes from tight to leaky strand type by introducing claudin-2 into Madin-Darby canine kidney I cells. J. Cell Biol..

[71] Weber C.R., Raleigh D.R., Su L., Shen L., Sullivan E.A., Wang Y., Turner J.R. (2010). Epithelial myosin light chain kinase activation induces mucosal interleukin-13 expression to alter tight junction ion selectivity. J. Biol. Chem..

[72] Rosenthal R., Gunzel D., Krug S.M., Schulzke J.D., Fromm M., Yu A.S. (2017). Claudin-2-mediated cation and water transport share a common pore. Acta Physiol..

[73] Rosenthal R., Gunzel D., Piontek J., Krug S.M., Ayala-Torres C., Hempel C., Theune D., Fromm M. (2020). Claudin-15 forms a water channel through the tight junction with distinct function compared to claudin-2. Acta Physiol..

[74] Rosenthal R., Milatz S., Krug S.M., Oelrich B., Schulzke J.D., Amasheh S., Gunzel D., Fromm M. (2010). Claudin-2, a component of the tight junction, forms a paracellular water channel. J. Cell Sci..

[75] Ayala-Torres C., Krug S.M., Schulzke J.D., Rosenthal R., Fromm M. (2019). Tricellulin effect on paracellular water transport. Int. J. Mol. Sci..

[76] Krug S.M., Amasheh S., Richter J.F., Milatz S., Günzel D., Westphal J.K., Huber O., Schulzke J.D., Fromm M. (2009). Tricellulin forms a barrier to macromolecules in tricellular tight junctions without affecting ion permeability. Mol. Biol. Cell.

[77] Staehelin L.A. (1973). Further observations on the fine structure of freeze-cleaved tight junctions. J. Cell Sci..

